# Effect of Various Warm-Up Protocols on Jump Performance in College Football Players

**DOI:** 10.2478/v10078-012-0086-5

**Published:** 2012-12-30

**Authors:** Jeffrey C. Pagaduan, Haris Pojskić, Edin Užičanin, Fuad Babajić

**Affiliations:** 1 College of Human Kinetics, University of the Philippines – Diliman, Philippines.; 2 Faculty of Physical Education and Sport, Tuzla University, Bosnia and Herzegovina.

**Keywords:** warm-up, static stretching, dynamic stretching, athletes, vertical jump

## Abstract

The purpose of this study was to identify the effects of warm-up strategies on countermovement jump performance. Twenty-nine male college football players (age: 19.4 ± 1.1 years; body height: 179.0 ± 5.1 cm; body mass: 73.1 ± 8.0 kg; % body fat: 11.1 ± 2.7) from the Tuzla University underwent a control (no warm-up) and different warm-up conditions: 1. general warm-up; 2. general warm-up with dynamic stretching; 3. general warm-up, dynamic stretching and passive stretching; 4. passive static stretching; 5. passive static stretching and general warm-up; and, 6. passive static stretching, general warm-up and dynamic stretching. Countermovement jump performance was measured after each intervention or control. Results from one way repeated measures ANOVA revealed a significant difference on warm-up strategies at F (4.07, 113.86) = 69.56, p < 0.001, eta squared = 0.72. Bonferonni post hoc revealed that a general warm-up and a general warm-up with dynamic stretching posted the greatest gains among all interventions. On the other hand, no warm-up and passive static stretching displayed the least results in countermovement jump performance. In conclusion, countermovement jump performance preceded by a general warm-up or a general warm-up with dynamic stretching posted superior gains in countermovement jump performance.

## Introduction

Team sports, like basketball, soccer, handball and American football consist of high intensive movements that include sprints, jumps, intermittent movement direction and speed changes with many acceleration and deceleration motions. These kinds of activities require proper body preparation in order to enable athletes to show their full physical potential, correspondingly to have as best as possible sport performance from the very beginning of a competition. A warm-up refers to the execution of physical exercise prior to the main activity in training or a competition (Hendrick, 1992). Coaches use different warm-up routines to facilitate the increase of body temperature, the acceleration of metabolism, and working capacities of heart and lungs of the athletes. A typical warm-up consists of aerobic activity (jogging, cycling, rope jumping etc.) followed by different kinds of stretching exercises (passive/active static stretching, or dynamic active stretching), but some use specific sport exercises or a combination of all above mentioned (Samson et al., 2012; Chaouachi et al., 2010; Vetter, 2007; Fletcher and Jones, 2004; Knudson et al., 2001). The stretching activity is generally promoted as a way of improving flexibility and preventing injuries, although existing evidence does not support this thesis (Magnusson and Renström, 2006). Dynamic and static stretching are the two major types of stretching interventions. Dynamic stretching involves the execution of a muscle group to a full range of motion without the help of an external force. On the other hand static stretching utilizes the assistance of an external force to achieve the full range of motion of a muscle group.

Previous studies revealed that static stretching led to reduced knee extensor power and jump performance compared to dynamic stretching (Costa et al., 2010; Hough et al., 2009; Yamaguchi and Ishii, 2005; Cornwell et al., 2002). However, when static stretching was incorporated with other dynamic activities (e.g. jogging), similar jump performance with dynamic stretching and dynamic activities was observed (Vetter, 2007; Chaouachi et al., 2010). Some authors reported deleterious effects of static stretching on sprint performance despite being combined with dynamic stretching or an aerobic warm-up (Sim et al., 2009; Winchester et al., 2008; Fletcher et al., 2007).

The purpose of this study was to determine the effects of different warm-up protocols on countermovement jump performance in college football players. It was hypothesized that countermovement jump performance preceded by dynamic actions would exhibit better results than static stretching or no warm-up.

## Material and methods

### Participants

Twenty-nine healthy male college football players (age: 19.4 ± 1.1 years; body height: 179.0 ± 5.1 cm; body mass: 73.1 ± 8.0 kg; % body fat: 11.1 ± 2.7) from the Tuzla University volunteered to participate in the study. They had a competitive experience of 6.5 ± 2.1 years and participated 10 hours per week in regular football training sessions and 3 hours per week in strength and conditioning training. A randomized control trial was applied to all the participants. None of the athletes had a history of neuromuscular disease or reported injuries for the past six months. The participants were informed about the purpose of the study, testing protocols, research benefits and potential risks. All of them signed a written informed consent. No dietary intervention was recommended in the study. The Ethical Committee of the Tuzla University approved the study with procedures conforming to the principles of the Declaration of Helsinki on human experimentation.

### Procedures

**Figure 1:**
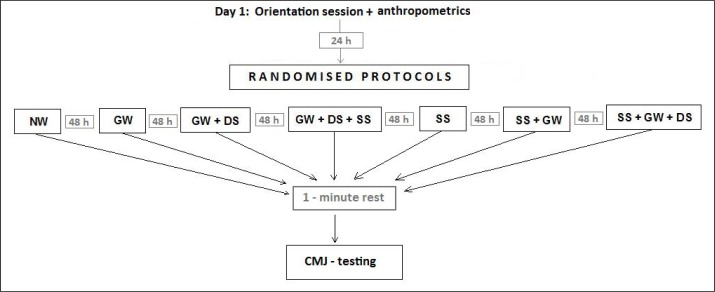
Experimental Protocols

All experiments were carried out at the Exercise Science Laboratory of Faculty of Physical Education and Sport, Tuzla University from 8 to 10 am. The experimental protocol design is displayed in Figure 1. Sessions were separated by 48 hours. Control and experimental groups were succeeded by countermovement jump trials after 1 minute of control or intervention. On Day 1 anthropometrics data were collected and the participants did not perform any warm-up activity. Day 2 was allotted to general warm-up performance. The general warm-up (GW) consisted of five minutes running at a preset pace. This was equivalent to 12 circles around an 86 m circumference area. In the first four circles, the participants had to run 30 seconds per circle. 25 seconds was required to finish the second four circles. In the last four circles, the participants had to run 20 seconds per circle. On Day 3, the participants performed GW and dynamic active stretching (DS). DS consisted of 7 exercises performed in 7 minutes (Table 1). Each exercise consisted of 2 sets of 20 seconds with a rest interval of 10 seconds between sets. The rest interval between exercises was 10 seconds.

The participants executed GW, DS and passive static stretching (SS) on Day 4. Seven static stretching exercises for 7 minutes were performed (Table 2). SS followed the same volume as in DS.

However, for unilateral stretching exercises, the first set was performed using the left limb followed by the right limb in the next set. All interventions involving SS were executed to the point of discomfort when stretching. SS was performed on Day 5. SS and GW protocol was administered during Day 6. Lastly, SS, GW and DS were executed by the participants on Day 7.

### Measures

With regard to anthropometrics data, body height (BH) was measured to the nearest 0.01m with a portable stadiometer (Astra scale 27310, Gima, Italy). Body mass (BM) and body fat percentage (%BF) were measured by a bioelectric body composition analyzer (Tanita TBF-300 increments 0.1%; Tanita, Tokyo, Japan).

Countermovement Jump Performance (CMJ) was assessed according to the protocol described by Bosco et al. (1983). Players were asked to start from an upright position with straight legs and with hands on hips in order to eliminate contribution of arm swing on jump height. The players executed a downward movement before the jump. Players performed a natural flexion before take-off. The participants were instructed to land in an upright position and to bend the knees on landing. Each player performed three maximal CMJ jumps, allowing three minutes of recovery between the trials. The highest score was used for analysis. The jumps were assessed using a portable device called the OptoJump System (Microgate, Bolzano, Italy) which is an optical measurement system consisting of a transmitting and receiving bar (each bar being one meter long). Each of these contains photocells, which are positioned two millimeters from the ground. The photocells from the transmitting bar communicate continuously with those on the receiving bar. The system detects any interruptions in communication between the bars and calculates their duration. This makes it possible to measure flight time and jump height during the jump performance. The jump height is expressed in centimeters.

### Statistical Analysis

Data are expressed as means and standard deviations. The Kolmogorov-Smirnov test was applied to test the data for normality. Interclass correlation coefficient (ICC) and coefficient of variation (CV) was calculated to assess reliability of the three vertical jump trails. One way repeated measures ANOVA was utilized to determine a significant difference in performance among the interventions. Effect size was established using eta squared. Bonferonni post hoc contrast was applied to determine pairwise comparison between interventions. Statistical significance was set at p<0.05. All statistical analyses were completed with the SPSS software statistical package (SPSS Inc., Chicago, IL; Version 14.0).

## Results

Warm-up protocols and CMJ height are displayed in Table 3. Results from one way repeated measures ANOVA showed a significant difference in warm-up strategies at F (4.07, 113.86) = 69.56, p < 0.001, eta squared = 0.72. Post hoc tests using Bonferroni correction determined that NW was significantly lower compared to GW, GW-DS, GW-DS-SS, SS-GW, SS-GW-DS at p = 0.001. GW elicited significant CMJ than GW-DS-SS and SS at p < 0.001. GW-DS posted better CMJ scores in comparison with GW-DS-SS, SS, and SS-GW-DS at p < 0.001. GW-DS-SS was significantly higher compared to SS but was lower than SS-GW-DS at p < 0.001. SS showed lower CMJ performance than SS-GW and SS-GW-DS at p < 0.001.

## Discussion

The purpose of this study was to investigate the effect of various warm-up protocols on countermovement jump performance. Results revealed that performance of GW and GW-DS posted superior gains in CMJ scores than other warm-up protocols examined in the study. Possible mechanisms in performance enhancement compared to other protocols include improvement in muscle stiffness and nervous system activation (Fletcher, 2010; Hough et al., 2009; Guissard and Duchateau, 2006; Kubo et al., 1999).

The reduced effect on jumping performance preceded by SS in this study agrees with the findings posted by Esposito et al. (2011). One possible mechanism that may explain the power output deficit of SS is the reduction in muscle stiffness (Esposito et al., 2011; Kubo et al., 2001; Wilson et al., 1992). SS may have led to more compliant series elastic components by decreasing actin - myosin overlapping and cross bridge formation. This produced a longer transmission of force to the insertion of the tendon. Another possible explanation is the reduction in the hysteresis of the muscle tendons (Kubo et al., 2002; Kubo et al., 2001). Hysteresis is the loss of energy as heat due to internal damping. The reduction of energy dissipation in the tissues after passive stretching may have caused the decreased tendon hysteresis in a similar vein. SS may have decreased muscle temperature and reduced nerve conduction velocity (Evans et al., 2002; Davies and Young, 1983; Bergh and Ekblom, 1979). Lastly, the stimuli in the static stretching protocol may have produced a level of neural inhibition that reduced the activation of motor units, thus resulting in lower countermovement jump performance (Costa et al., 2010; Hough et al., 2009; Cornwell et al., 2002).

In this study, there was a non-significant difference in CMJ between NW and SS. However, SS showed higher CMJ scores than NW. This finding may imply that performance of SS instead of NW is favorable to CMJ. In another light, when SS is combined with GW and DS, CMJ deficit is reduced. The existence of better CMJ when SS is applied pre GW and DS than post GW and DS suggests that mechanical and neural responses similar to SS may be reduced if SS is succeeded by dynamic actions. This finding coincided with the study administered by Holt and Lambourne (2008) but contradicted other studies (Chaouachi et al., 2010; Vetter, 2007). The study of Chaouachi et al. (2010) involved elite or national level student-athletes from different sports.

On the other hand, the participants in Vetter’s study (2007) included physically active and recreationally active individuals. Both studies suggest that variations in physiological demands and a physical activity level may influence the effect of integrating SS with GW and DS in CMJ performance.

In conclusion, the use of warm-up protocols may produce mechanical and neural responses that may affect countermovement jump performance. In this study, performing SS and NW before CMJ showed significant reductions in CMJ. Also, SS following dynamic warm-up interventions inhibited the jump performance in collegiate football athletes. It is interesting that the application of dynamic active stretching conducted after passive stretching could not recover negative effects of passive stretching. Although the study provided evidence that may assist practitioners in designing warm-up strategies in performance settings, certain limitations should be noted. The study is only limited to an acute finding using CMJ performance only. Future studies should warrant the use of other performance measures in longer time settings. Also, the experimental protocols failed to quantify physiological measures (e.g. heart rate, temperature) which may be helpful in understanding the current findings. Finally, the results in the study are specific to the participants chosen for the experiment. Caution should be exercised in generalizing the effects across other population.

## Figures and Tables

**Table 1:** Dynamic Stretching Exercises

Straight Leg March
Butt Kicks
Carioca
High Knees
Reverse Lunge with Twist
Power Shuffle (Step Slide)
Jogging with Squats

**Table 2:** Static Stretching Exercises

Standing Quadriceps Stretch
Standing Calf Stretch
Standing Hamstring Stretch
Single Leg Straddle
Inverted Hurdler's Stretch
Lying Single Knee to Chest
Seated Cross-Legged Gluteus Stretch

**Table 3:** Warm-Up Protocols and CMJ Height (mean, standard deviation)

Warm-Up Protocols	CMJ Height (cm)	ICC	CV
No Warm-Up	33.7, 3.8	0,87	0,11
General Warm-Up	38.0, 4.3	0,91	0,11
General Warm-Up, Dynamic Stretching	39.1, 4.8	0,95	0,12
General Warm-Up, Dynamic Stretching, Passive Static Stretching	36.2, 4.7	0,93	0,13
Passive Static Stretching	34.3, 4.1	0,84	0,12
Passive Static Stretching, General Warm-Up	37.4, 4.2	0,92	0,11
Passive Static Stretching, General Warm-Up, Dynamic Stretching	38.2, 4.3	0,9	0,11
